# Transcriptional Network Growing Models Using Motif-Based Preferential Attachment

**DOI:** 10.3389/fbioe.2015.00157

**Published:** 2015-10-12

**Authors:** Ahmed F. Abdelzaher, Ahmad F. Al-Musawi, Preetam Ghosh, Michael L. Mayo, Edward J. Perkins

**Affiliations:** ^1^Biological Networks Laboratory, Department of Computer Science, Virginia Commonwealth University, Richmond, VA, USA; ^2^Thi Qar University, Al-Nasiriyah, Iraq; ^3^Environmental Laboratory, US Army Engineer Research and Development Center, Vicksburg, MS, USA

**Keywords:** motif, degree distribution, power-law, attachment kernel, transcriptional network

## Abstract

Understanding relationships between architectural properties of gene-regulatory networks (GRNs) has been one of the major goals in systems biology and bioinformatics, as it can provide insights into, e.g., disease dynamics and drug development. Such GRNs are characterized by their scale-free degree distributions and existence of network motifs – i.e., small-node subgraphs that occur more abundantly in GRNs than expected from chance alone. Because these transcriptional modules represent “building blocks” of complex networks and exhibit a wide range of functional and dynamical properties, they may contribute to the remarkable robustness and dynamical stability associated with the whole of GRNs. Here, we developed network-construction models to better understand this relationship, which produce randomized GRNs by using transcriptional motifs as the fundamental growth unit in contrast to other methods that construct similar networks on a node-by-node basis. Because this model produces networks with a prescribed lower bound on the number of choice transcriptional motifs (e.g., downlinks, feed-forward loops), its fidelity to the motif distributions observed in model organisms represents an improvement over existing methods, which we validated by contrasting their resultant motif and degree distributions against existing network-growth models and data from the model organism of the bacterium *Escherichia coli*. These models may therefore serve as novel testbeds for further elucidating relationships between the topology of transcriptional motifs and network-wide dynamical properties.

## 1 Introduction

The dynamics of complex networks are derived using graph theoretical measurements that are deduced from the topology of the network entities and their relationships. For example, science collaboration networks are portrayed using nodes that represent scientists or authors, and links that connect pairs of nodes that coauthored an article (Albert and Barabási, 2002). Unlike engineered networks such as wireless sensor networks (Li et al., 2012) and airline transportation networks (Bensong et al., 2010), science collaboration networks fall under the “small world” category of complex networks due to their smaller average over the ensemble of shortest connected paths through a network. Networks subscribing to the same category, such as the World Wide Web, cell structures networks, protein–protein interaction networks, the Internet, and infectious disease networks have all been analyzed for path lengths, cluster formations, degree distributions, and evolutionary patterns (Albert et al., 1999; Albert and Barabási, 2002; Alm and Arkin, 2003; Alon, 2003; Dorogovtsev and Mendes, 2003; Newman, 2003; Barabasi and Oltvai, 2004; Wang, 2004; Meyers et al., 2005). Gene regulatory networks (GRNs) also belong to this category. Understanding the dynamical consequences implied by the architecture of GRNs has been one of the major goals in systems biology and bioinformatics, as it can provide insights into, e.g., disease dynamics and drug development (Margolin et al., 2006; Faith et al., 2007). In gene-regulatory networks, the nodes portray products of genes or transcription factor proteins within a cell, and a set of directed bonds which each denote pairs of nodes that interact by altering the activity of the target gene (Shmulevich and Dougherty, 2010) parameterized by the biological processes of translation and transcription (Feng et al., 2007). Unlike engineered communication networks [as in Ghosh et al. (2005)], GRNs exhibit a unique withstanding property – a phenomenon known as “Biological Robustness” (Kitano, 2004, 2007), which describes an ability of individual genes to adapt to and potentially resist disturbances to gene activity based, in part, on their connectivity to other genes of the network (Prill et al., 2005). Such a useful property could be potentially exploited to design engineered networks with similar communication properties (Ghosh et al., 2011; Kamapantula et al., 2012, 2014 and Kamapantula et al., under review).

Robustness in the expression patterns may arise from feed-back-based regulatory loops or arrangements between various repetitive subnetworks (Kauffman, 1993). This begs the question of whether such robustness can be attributed to some statistically significant GRN subnetwork, termed as transcriptional motifs (Alon, 2007). Transcriptional motifs may represent “building blocks” of many complex networks (Milo et al., 2002) (including GRNs), as they appear more commonly in GRNs than observed in randomized versions of these networks (Milo et al., 2002) – i.e., networks with the same number of nodes, links, and degree distribution as the principle network, but different overall topology. Although much consideration has been focused toward unfolding individual properties of transcriptional motifs, both theoretically (Magnan and Alon, 2003) and experimentally (Wu and Rao, 2010), little remains known regulating their patterns of interactions to the biological mechanisms of natural evolution.

**Figure 1:**
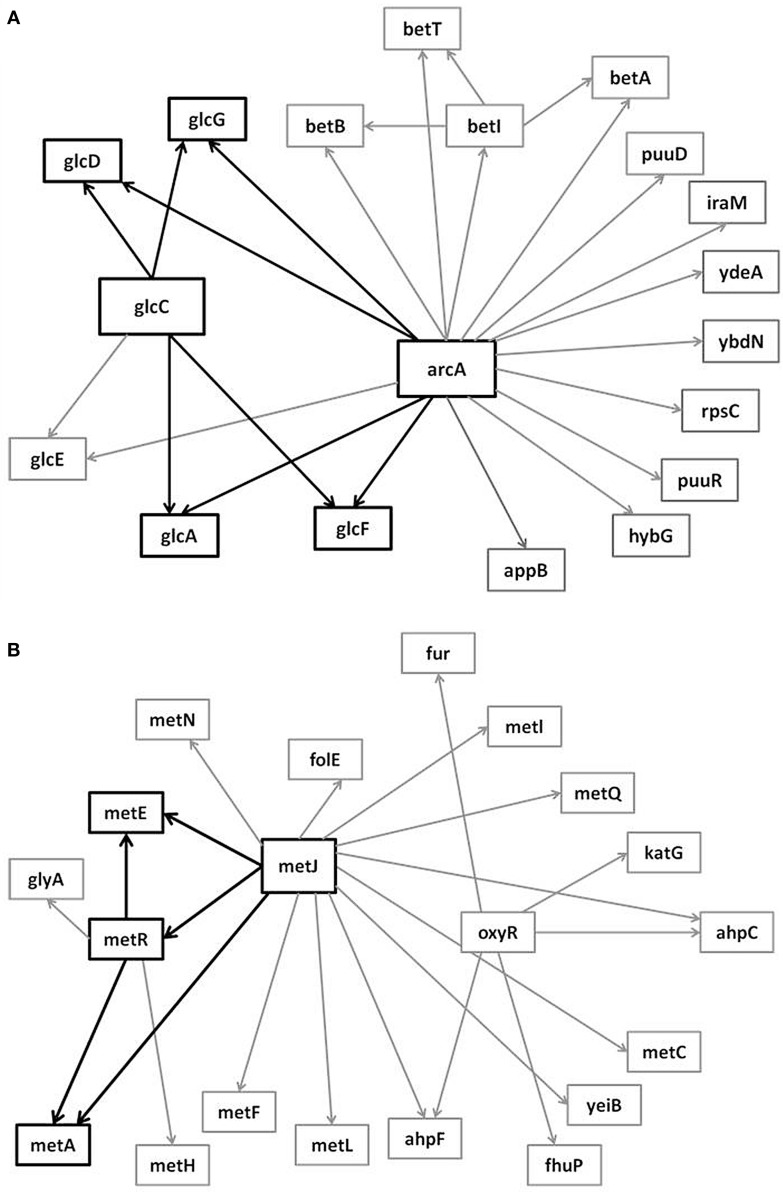
**Embedded within sample GRN subgraphs of *E. coli*, the topological representation of (A) bifans**. Here, transcription factors arcA and glcC co-regulate glcD and glcG. On the other hand, **(B)** the feed-forward loop constitutes a transcription factor (such as metJ) that regulates both a gene (metE) and another transcription factor (metR). The regulated transcription factor co-regulates the same gene (metR → metE).

In the supplementary materials of Milo et al. (2002), the authors enumerate all possible 3–6 node transcriptional motifs. Among the most common transcriptional motifs observed in GRNs of the model bacterium *Escherichia coli* (herein *E. coli*) and the baker’s yeast *Saccharomyces cerevisiae* (herein labeled Yeast), are feed-forward loops (FFLs) and bifans (BFs), which can be observed natively in Figures 1A,B. An FFL is hierarchically composed of three genes, a top-level “father” gene that regulates two “child” genes, wherein one of the child genes regulates the other. This specific topology allows for interesting dynamical consequences, such as pulses, signal delays, and irreversible speed-ups (Magnan and Alon, 2003). By contrast, BFs constitute four genes, two of which simultaneously regulate the other two; these motifs have been reported as constituents of dense overlapping regulons in the GRN “backbone” responsible for vital life functions, such as nutrient metabolism and bio-synthesis (Alon, 2007).

**Figure 2:**
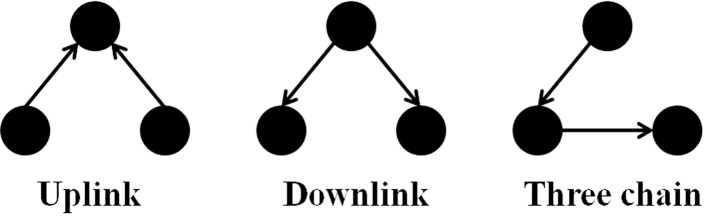
**The three-node two-edge motif substructures**.

It is notable to point out that many motifs are a product of the coupling between the subnetworks illustrated in Figure 2: the uplink, the downlink, and the three chain. For instance, a BF can be viewed as two downlinks coupled by sharing both child genes, while an FFL can be viewed as an uplink or a downlink sharing all three genes with a three-chain. Moreover, we have conducted computational analysis to estimate the percentages of the gene-regulatory interactions that participate in these components for an *E. coli* GRN. We observed that 54.7% of interactions are involved with FFLs, 82% with BFs, 99.4% by downlinks, 83.9% by uplinks and 78.3% by three-chains. Given these data for *E. coli*, we hypothesize that downlinks represent a primary component in the evolution of GRN topology. Despite that the impacts of motif-coupling on the functionality of GRNs remain largely mysterious, some results have been reported in this particular area. For example, investigations of gene coupling for different motif patterns have been conducted using mathematical modeling of transcription and translation in order to reveal substructure functionalities (Yung-keun and Kwang-hyun, 2007; Kim et al., 2008; Wu and Rao, 2010). Additionally, experiments have revealed that bacteria can endure a great deal of regulatory interaction rewiring via manipulation of protein-binding DNA sequences (Isalan et al., 2008).

To further understand how transcriptional motifs “interact” via regulatory bonds, we have previously studied how the individual genes of *E. coli* are distributed through the FFLs of its GRN (Mayo et al., 2012). There we contrasted node-motif distributions of *E. coli* with “randomized” networks constructed node-by-node via a preferential attachment algorithm that leveraged both linear and non-linear attachment kernels (Krapivsky et al., 2000). This modified preferential attachment algorithm resulted in FFL abundances that compared well to the overall GRN of *E. coli*; however, fidelity of the motif participation distribution of the nodes in the generated network was very low when compared with that from *E. coli*. In this paper, we extend this prior algorithm based on the following two criteria:

1. Our modified preferential attachment algorithm was oblivious to the distinction of the two different types of nodes in transcriptional networks: genes and transcription factors (TFs). Since transcriptional networks only allow TF-to-TF and TF-to-gene edges, a distinction between these biological classes that restricts allowed bonds may improve fidelity of the “grown” networks to that from *E. coli* or other GRNs.
2. The previous algorithm considered attachment of one node at a time to the substrate network for growth following the general premise of preferential attachment. However, this failed to generate the correct FFL motif distribution of the nodes in the grown network as compared to the GRN of *E. coli*. In this paper, we consider the attachment of an entire downlink motif at a time using a preferential attachment methodology. One or more of the three nodes of the incoming downlink may be shared with selected nodes in the substrate network resulting in the growth of the network by one (if two vertices are shared between the incoming downlink and substrate network) or two nodes (if one vertex is shared between the incoming downlink and substrate network) or zero nodes (if all three vertices of the incoming downlink are shared with corresponding three vertices in the substrate) at a time. The motivation for a downlink-based preferential attachment model stems from an observation that 99.4% of the nodes in the GRN of *E. coli* participate in downlinks.

## 2 Related Works

Algorithms [e.g., Mayo et al. (2012)] that generate scale-free directed networks aim to mimic a target networks’ topological properties, and are useful for understanding processes that govern dynamical formation of many complex networks. Features considered in our previous analysis were the distributions of the in-, out-, cumulative degrees, and the participation of genes in FFLs (see Methods and Materials for details) of the largest connected component of *E. coli*’s transcriptional-regulatory network. We consider the same features in the analysis of the proposed algorithm in this paper for comparing the generated and target networks, except for gene participation, where we consider the genes that participate in downlinks only, but not FFLs (Mayo et al., 2012). A brief description of the modified preferential attachment algorithm from Mayo et al. (2012) follows.

A candidate node in the existing substrate network of *n* nodes at given time – i.e., the network resultant from the sum total of all previous attachment steps – is denoted with subscript *i* (1 ≤ *i* ≤ *n*). The probability for this candidate node to be connected to an external (incoming) node with a directed edge incident on the external node is given by *A*(*K_i_*, *R_i_*), wherein *K_i_* and *R_i_* denote, respectively, its out- and in-degrees. The probability that a link is projected from the external node onto the candidate node is given by *B*(*K_i_*, *R_i_*). The probabilities of all the candidate nodes are normalized to form attachment kernels that determine whether a link is to be considered (Krapivsky et al., 2000). The formulae for three different attachment kernels considered in Mayo et al. (2012) are given in Table 1.

**Table 1:** **Attachment kernels used here to “grow” networks (Mayo et al., 2012)**.

Functional type	Attachment Kernels	
	*A*(*K_i_*, *R_i_*)	*B*(*K_i_*, *R_i_*)
Linear	$\frac{K_{i}}{\sum_{i=1}^{n}K_{i}}$	$\frac{R_{i}}{\sum_{i=1}^{n}R_{i}}$
Power-law	$\frac{K_{i}^{0.8}}{\sum_{i=1}^{n}K_{i}^{0.8}}$	$\frac{R_{i}^{0.8}}{\sum_{i=1}^{n}R_{i}^{0.8}}$
Sigmoid	$\frac{K_{i}}{\sum_{i=1}^{n}\left(K_{i}+R_{i}\right)}$	$\frac{R_{i}}{\sum_{i=1}^{n}\left(K_{i}+R_{i}\right)}$

The algorithm from Mayo et al. (2012) allows for multiple links to be placed per attachment step; therefore, it was necessary to consider nucleation of the network from a connected 8-node candidate network at *t* = 0 to avoid null attachments. A candidate node is always selected at random if it has not been selected before during a single attachment. Next, a random number, *d*, is drawn uniformly from the interval [0,1]. If the condition *d* ≤ *A*(*K_i_*, *R_i_*) is satisfied, an outgoing link from the candidate node is connected to the external node. The process is then repeated for an outgoing link originating from the external node that connects to a candidate node, provided the probability satisfies *d* ≤ *B*(*K_i_*, *R_i_*). This process is repeated *m_i_* − 1 times wherein *m_i_* is an integer drawn at random from an exponential probability distribution:

(1) $$\rho \left(m_{i}\right)=\left(f^{\frac{1}{1-m_{0}}}-1\right)f^{-m_{i}∕\left(1-m_{0}\right)},$$

wherein *f*  = 0.25 and *m*_0_ ∈ {2, 3, 4} (Mayo et al., 2012).

The attachment mechanism of this algorithm is similar to that of the Barabási-Albert model (Barabási and Albert, 1999) (BA), in that it preserves the phenomenon of “the rich get richer and the poor get poorer.” For instance, a node having relatively large number of outgoing links will probably continue to increase its out-degree during attachments together with a smaller chance of connecting nodes with fewer incoming links. However, early versions of the BA model did not account for the directionality of the links; while it could not be expected to topological properties of biological networks with high fidelity, it was very successful in capturing many of their qualitative features, such as the scale-free degree distribution. By contrast, other models, such as the duplication divergence (DD) model suggested by Vázquez et al. (2003), have been used to generate model biological networks, which was later extended in Chung et al. (2003). The DD model was designed based on the fact that proteins/genes evolve through duplication followed by spasmodic mutations. However, only very few of the networks grown show resemblance to their final target structures in terms of degree distributions. The modified preferential attachment algorithm in Mayo et al. (2012) reflects a first attempt to create a directed biological network growing algorithm capable of preserving the abundance of FFLs in “grown” random networks with reasonable accuracy as compared to the largest connected component of *E. coli*’s transcriptional network.

## 3 Materials and Methods

### 3.1 Transcriptional *Network Datasets*

To evaluate the fidelity of artificially constructed networks, we sampled subnetworks from the entire body of the *E. coli* transcriptional network, herein referred to as “target networks.” As mentioned above, we defined two types of nodes arranged hierarchically in these GRNs, classified as either (a) genes or (b) transcription factors, and defined such that genes reflect a regulatory terminus wherein they do not regulate other nodes (i.e., have no outgoing links), and transcription factors are nodes that regulate genes. Consequently, there are three possibilities for the class of nodes that constitute a downlink motif:

1. three transcription factors (herein TTT);
2. a transcription factor regulating two genes (herein TGG); or
3. a transcription factor that regulates another transcription factor and a gene (herein TTG).

All transcriptional interactions of *E. coli* GRNs have been validated experimentally (Shen-Orr et al., 2002), and target networks have been rendered using GeneNetWeaver (Schaffter et al., 2011) – a bioinformatics software originally designed to evaluate the accuracy of network inference algorithms. GeneNetWeaver provides options for sampling subnetworks from the GRNs of both *E. coli* and *S. cerevisiae*. The *E. coli* network supported by GeneNetWeaver is composed of 23 disjoint components together encompassing 1,565 genes and 3,758 links. Here, we focus our investigations on connected GRNs; hence, we consider *E. coli*’s largest connected component (LCC), which itself contains 1,477 nodes and 3,671 links. Moreover, our analyses do not account for the effects of self-loops associated with transcription factors. For simplicity, we have removed them from the target networks considered here.

### 3.2 Vertex-Based Motif Networks and Downlink Coupling

Conventional preferential attachment models estimate the attachment probability from the degree of single candidate nodes in the target networks. However, to conceptualize a downlink-based preferential attachment method, which is a collection of nodes, we must first identify a way to express a downlink motif from the substrate network into a single, effective “lumped” node.

**Figure 3:**
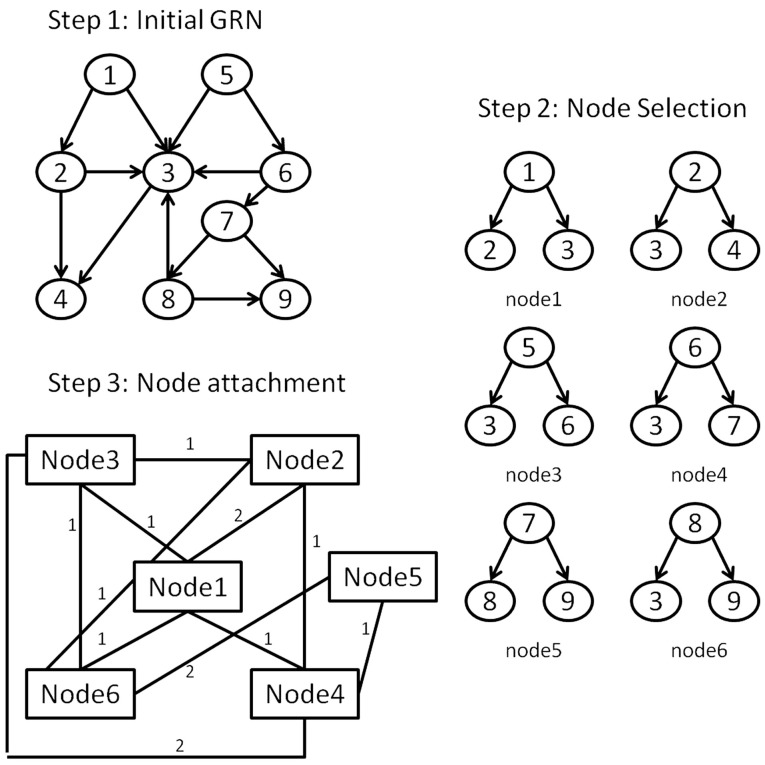
**The steps for forming the VMN from a GRN: (Step 1) An initial GRN is considered**. (Step 2) A list of the downlink structures is derived from the GRN giving each downlink structure its unique id. (Step 3) Each downlink’s constituent nodes are contrasted with every other downlink’s nodes. Downlinks form topological interactions in the VMN if they have at least one common node. The strength of the interaction is equivalent to the number of shared nodes between the corresponding downlinks.

To achieve this we propose to apply a network transformation to the *E. coli* LCC, defined so that each node of the transformed network represents a downlink derived from the LCC; downlink “nodes” are connected to others with edges weighted by the number of nodes shared between the two downlink motifs. For example, two downlink motifs that share a single node would equate with two nodes connected by a single link of unit weight. Herein we term such a resultant network, a vertex-based motif network (VMN). An illustration of this graph transformation is shown in Figure 3. VMNs are therefore manifestly undirected networks. Although *E. coli* is sparse (Genio et al., 2011), its equivalent VMN contains many more nodes due to the approximately 278,000 downlinks supported in the network, most of which share nodes due to the hierarchical nature of the *E. coli* GRN. Therefore, it’s VMN is dense.

**Figure 4:**
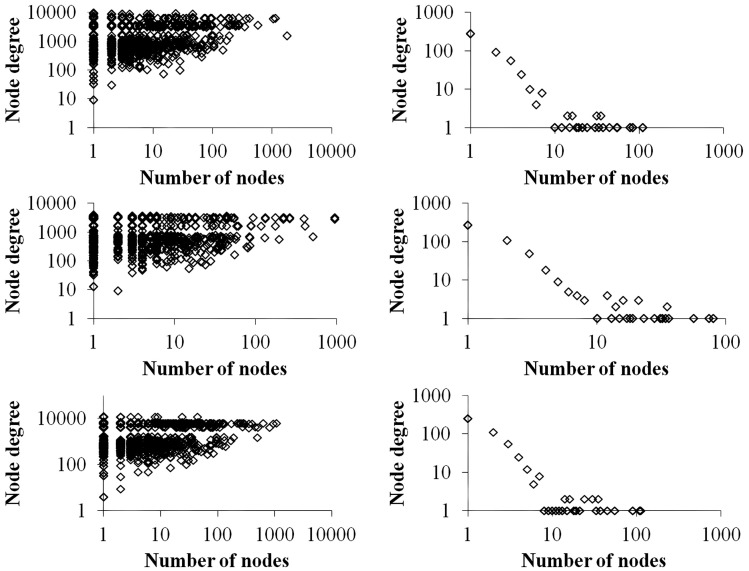
**A plot of the number of nodes (vertical axis) vs. the cumulative degrees (horizontal axis) of VMNs (left) as compared to their respective GRNs (right)**.

Figure 4 contrasts differences in the total degree distributions of three sample GRN subnetworks of sizes *n* = 500 (right panels) with their corresponding VMNs (left panels). Some VMNs reached as much as 400-fold the number of nodes as their original subnetwork. Finally, we note that degree distributions exhibited by VMNs indicate an absence of correlation in the abundance of shared vertices among downlink motifs.

### 3.3 Data Representation

Computationally, we have represented GRNs and VMNs using square matrices, respectively, labeled *G* and *V*. A GRN link from node *j* and incident on node *k* is represented by *G_jk_* = 1, and the absence of such connection is represented by *G_jk_* = 0, similar to an adjacency matrix. Because GRN links carry no weight, the matrix *G* may only hold values of 0 and 1. In *G* of size *n*, the downlink count *S_DL_* can be determined mathematically using the equation:

(2) $$S_{\mathrm{DL}}=\frac{1}{2}\overset{n}{\underset{a=1}{\sum }}\overset{n}{\underset{b=1}{\sum }}\overset{n}{\underset{c=1}{\sum }}\left[G_{\mathrm{ab}}\cap G_{\mathrm{ac}}\right].$$

However, *V* differs from *G* in that it is symmetric with elements given by the weights 0, 1, 2, and 3, depending on the number of vertex overlaps between one downlink and another. Therefore, *V_lm_* = *V_ml_* = 0 if downlinks *l* and *m* do not share any nodes, *V_lm_* = *V_ml_* = 1 if downlinks *l* and *m* share one node, and so on.

### 3.4 Algorithm for Network Growth

A subnetwork of a target network, termed a “substrate,” accumulates one downlink per attachment step. Table 2 illustrates possible downlink-to-downlink attachments, as based on the number of vertices shared between a candidate and incoming downlink motif (DL). In order to determine the appropriate attachment, the following steps are considered.

**Table 2:** **Every type of potential downlink-to-downlink attachment**.

Category	Pattern id	Pattern graph	Attachment description	Applicable DL–DL combinations
One node attachment	P1	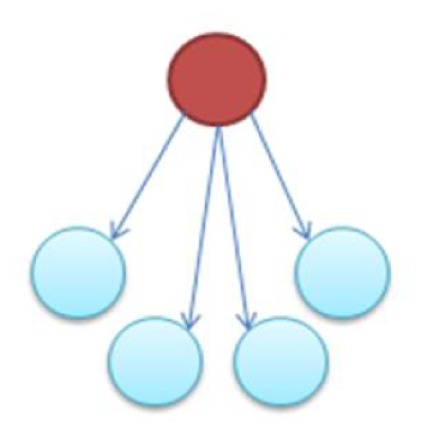	Root TF coupling	TGG–TGG, TTG–TGG, TTT–TGG, TGG–TTG, TTG–TTG, TTT–TTG, TGG–TTT, TTG–TTT, TTT–TTT
	P2	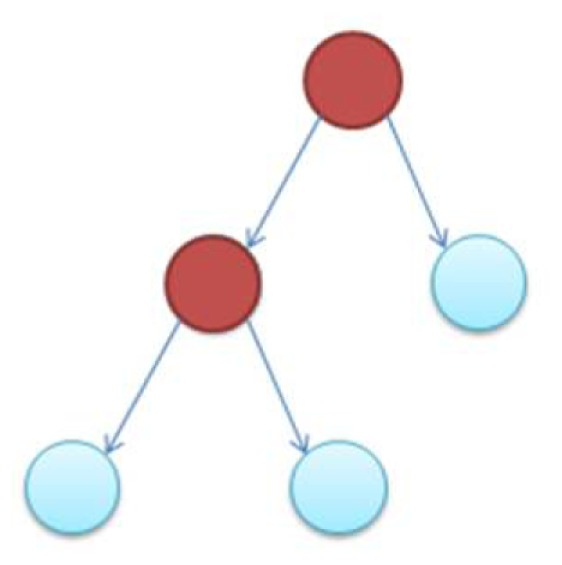	Leaf TF to root TF coupling	TTG–TGG, TTT–TGG, TGG–TTG, TTG–TTG, TTT–TTG, TGG–TTT, TTG–TTT, TTT–TTT
	P3	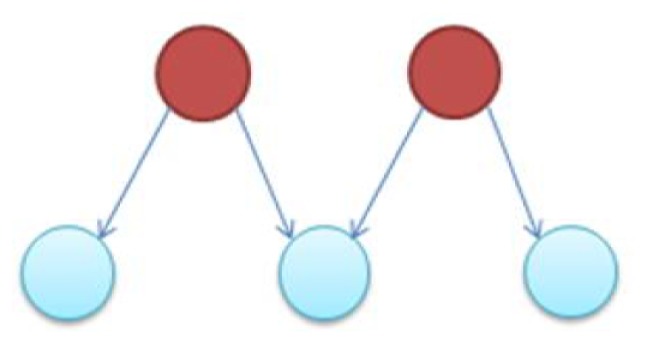	Leaf gene coupling	TGG–TGG, TTG–TGG, TGG–TTG, TTG–TTG, TTT–TTG, TTG–TTT, TTT–TTT
Two node attachment	P4	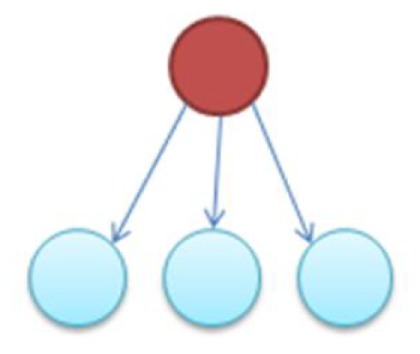	(1) Root TF coupling and (2) one leaf gene coupling	TGG–TGG, TTG–TGG TGG–TTG, TTG–TTG, TTT–TTG, TTG–TTT, TTT–TTT
	P5	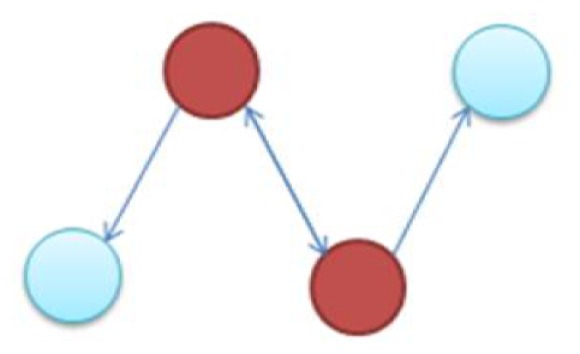	(1) Root TF couples with leaf TF, and (2) one leaf TF couples with root TF	TGG–TTG, TTG–TTG, TTT–TTT
	P6	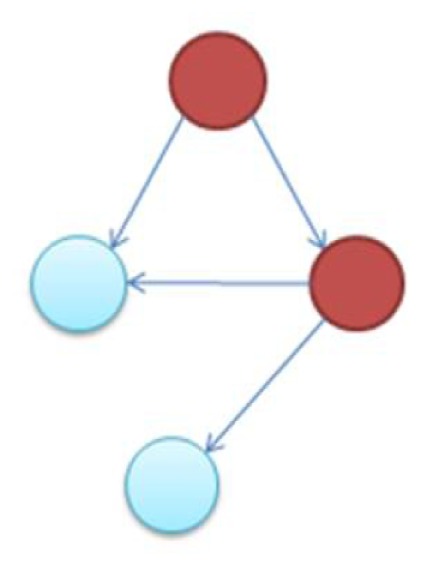	(1) Leaf TF couples with root TF, and (2) one leaf gene couples with leaf node	TTG–TGG, TGG–TTG, TTG–TTG, TTT–TTG, TTT–TTT
	P7	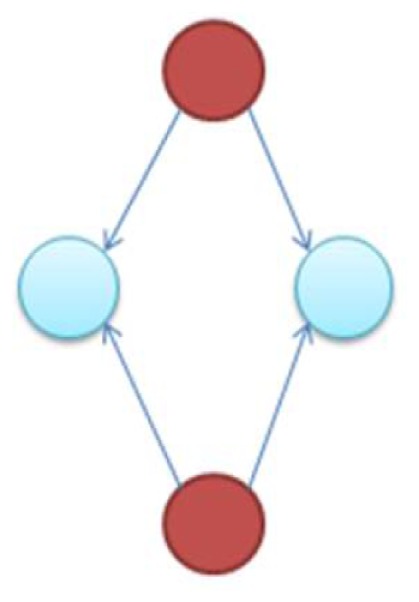	(1) Leaf gene couples with leaf gene, and (2) one leaf gene couples with leaf gene	TGG–TGG, TTG–TTG, TTT–TTT
Three node attachment	P8	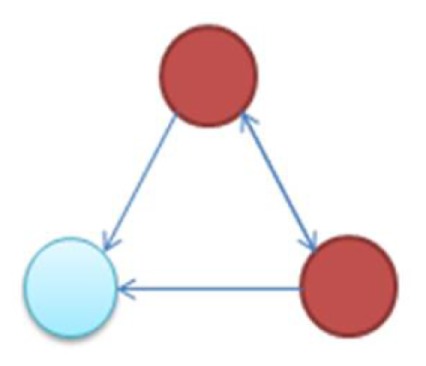	(1) Root TF couples with leaf TF, and (2) one leaf TF couples with root TF, and (3) one leaf gene couples with leaf gene	TTG–TTG, TTT–TTT

#### 3.4.1 Step 1 – Determine Candidate Downlink Type

In order to select an existing downlink from the substrate network as a candidate for attachment, its type needs to be specified. We denote the sums of the three downlink types as *N_TGG_*, *N_TTG_*, and *N_TTT_*, such that

(3) $$S_{\mathrm{DL}}=N_{\mathrm{TGG}}+N_{\mathrm{TTG}}+N_{\mathrm{TTT}}.$$

Using Eq. 3, the probability that a selected candidate downlink is of type TGG, TTG, or TTT is determined by, $P_{\mathrm{TGG}}=\frac{N_{\mathrm{TGG}}}{S_{\mathrm{DL}}}$, $P_{\mathrm{TTG}}=\frac{N_{\mathrm{TTG}}}{S_{\mathrm{DL}}}$, and $P_{\mathrm{TTT}}=\frac{N_{\mathrm{TTT}}}{S_{\mathrm{DL}}}$ in that order. These probabilities are later used as selection kernels to determine the type of candidate downlink. A random number, *r*_1_, is generated with uniform probability on the interval *r*_1_ ∈ [0,1]. If 0 ≤ *r*_1_ < *P_TGG_*, a TGG downlink is considered as a candidate for attachment. If *P_TGG_* ≤ *r*_1_ < *P_TGG_* + *P_TTG_*, a TTG downlink is considered. Otherwise a TTT is considered for attachment.

#### 3.4.2 Step 2 – Selection of Candidate Downlink

A VMN is created from the downlinks subscribing to the type selected in Step 1 and the preferential attachment mechanism is employed (Barabási and Albert, 1999). A random downlink *l* is picked with uniform probability, and its degree centrality is calculated as follows:

(4) $$C_{l}=\frac{\sum_{a=1}^{t-1}V_{\mathrm{la}}}{\sum_{a=1}^{t}\sum_{b=1}^{t}V_{\mathrm{ab}}},$$

wherein *t* represents the total number of downlinks in the VMN. Next, a random number 0 ≤ *r*_2_ < 1, is compared with *C_l_* such that if *r*_2_ < *C_l_*, *l* is selected as a candidate downlink. On the other hand, if the condition is not satisfied another downlink is picked at random and the process is repeated.

#### 3.4.3 Step 3 – The Type of Incoming Downlink

Incoming downlinks may be either of the three downlink types, generated at random with uniform probability.

#### 3.4.4 Step 4 – The Number of Shared Nodes

A similar strategy to that of Step 1 is implemented, except that the probability distribution depends on the number of shared nodes between pairs of downlinks and not the number of each type of downlink. There are *S_pair_* = *S_DL_* (*S_DL_* *–* 1)/2 total cases of downlink pairs sharing nodes, each of which can share 0, 1, 2, or 3 nodes. Since our model does not account for disjoint components, we ignore the cases where downlink pairs share no nodes. We denote the number of pairs sharing 1, 2, and 3 nodes as *N_s_*_1_, *N_s_*_2_, and *N_s_*_3_, respectively. Consequently the probabilities for node sharing can be determined by $P_{s1}=\frac{N_{s1}}{\left(N_{s1}+N_{s2}+N_{s3}\right)}$, $P_{s2}=\frac{N_{s2}}{\left(N_{s1}+N_{s2}+N_{s3}\right)}$, and $P_{s3}=\frac{N_{s3}}{\left(N_{s1}+N_{s2}+N_{s3}\right)}$. Next a third random variable 0 ≤ *r*_3_ ≤ 1 will be compared with the ranges (0, *P_s_*_1_), (*P_s_*_1_, *P_s_*_1_ + *P_s_*_2_), and (*P_s_*_1_ + *P_s_*_2_, *P_s_*_1_ + *P_s_*_2_ + *P_s_*_3_), respectively, to determine the number of shared nodes as was done in Step 1.

#### 3.4.5 Step 5 – The Attachment Pattern

Knowing the candidate downlink, the type of incoming downlink and the number of nodes to be shared (or overlapped), we can use Table 3 to proceed with an attachment. For example, having selected a candidate TGG, an incoming TTG, which will share two nodes, from Table 3 we are only allowed to proceed with three attachment patterns {P4, P5, P6}. Each pattern is given an equal probability of being chosen (here 1/3). A process similar to the random number generated in Steps 1 and 4 is used to determine which pattern will be chosen.

**Table 3:** **Applicable downlink to downlink attachments for a given candidate downlink, incoming downlink, and number of vertex overlaps**.

	DL–DL combination	Applicable patterns	DL–DL combination	Applicable patterns	DL–DL combination	Applicable patterns
One node attachment	TGG–TGG	{P1, P3}	TTG–TGG	{P1, P2, P3}	TTT–TGG	{P1, P2}
	TGG–TTG	{P1, P2, P3}	TTG–TTG	{P1, P2, P3}	TTT–TTG	{P1, P2, P3}
	TGG–TTT	{P1, P2}	TTG–TTT	{P1, P2, P3}	TTT–TTT	{P1, P2, P3}
Two node attachment	TGG–TGG	{P4, P7}	TTG–TGG	{P4, P6}	TTT–TGG	NA
	TGG–TTG	{P4, P5, P6}	TTG–TTG	{P4, P5, P6, P7}	TTT–TTG	{P4, P6}
	TGG–TTT	NA	TTG–TTT	{P4}	TTT–TTT	{P4, P5, P6, P7}
Three node attachment	TGG–TGG	NA	TTG–TGG		TTT–TGG	NA
	TGG–TTG	NA	TTG–TTG	{P8}	TTT–TTG	NA
	TGG–TTT	NA	TTG–TTT		TTT–TTT	{P8}

### 3.5 Maximum Likelihood Estimation

A key task in the analysis of many biological networks is to estimate the exponent of a power-law type degree distribution (Clauset et al., 2009). To assess the performance of our proposed algorithm, we evaluated the following relationships:

- in-degree distribution, viewed as a plot of the different in-degrees against the number of nodes possessing those in-degrees;
- out-degree distribution, viewed as a plot of the different out-degrees against the number of nodes that posses those out-degrees;
- total-degree distribution, taken as a plot of the different total-degrees against the number of nodes that posses those total-degrees; and
- distribution of genes participating in downlinks, which is the relationship between the number of downlinks, vs. the number of nodes that participate in all the different downlinks of the network.

A curve-fitting methodology is commonly used to estimate the fitted parameters; however, a least squares-based optimization algorithm may not accurately determine whether the data are power-law distributed (Hoogenboom et al., 2006; Clauset et al., 2009). To address this issue, Hoogenboom et al. (2006) presented a maximum likelihood estimation-based approach to determine whether a distribution follows a power-law. We used this method to compare best-fit values of power-law exponents for target networks with the substrate networks grown using our proposed algorithm.

## 4 Results and Discussion

### 4.1 Fidelity of the Downlink-Based Preferential Attachment Mechanism

We extracted five different target networks of 100 nodes from the *E. coli* LCC using the GeneNetWeaver software in the manner explained above. We extracted substrate subnetworks upon which to “grow” new networks from these target networks of relative sizes equal to 10, 20, 30, and 40 nodes. We sampled five substrates of each size, resulting in a total of 20 substrate subnetworks per target network derived from the *E. coli* LCC. Each substrate network was grown to a size of 100 nodes using two algorithms: (i) the attachment kernel (linear, power-law, and sigmoidal) method as presented in Mayo et al. (2012) and (ii) the downlink-based attachment mechanism explained above.

For networks generated using the downlink-based preferential attachment mechanism, we calculated the three types of downlink attachment probabilities in two ways. In the first method, termed “target attachment,” values for the fraction of downlinks of each type, *P_TGG_, P_TTG_*, *P_TTT_*, and fractions of downlinks that share one ($P_{s_{1}}$), two ($P_{s_{2}}$), and three ($P_{s_{3}}$) vertices were all calculated from the target networks derived from the *E. coli* LCC. This method is biased, given that we must use the structure of the biological networks to inform that of the “grown” networks. The second method, termed “substrate attachment,” calculates the same probabilities as the first method, but iteratively from the current state of the grown network. This method is unbiased, in the sense that it is ignorant of the final topology of the target network.

Degree distributions of the “grown” networks were fitted to the data using a power-law equation, and each of the two methods was compared individually to the fitted exponents of the biological networks as a measure of their fidelity. Exponents, γ, were estimated not only for in-, out-, total degree distributions (Table 4) but also for distributions relating the participation of nodes in downlink substructures (Table 5). A lower value for the difference in fitted exponents suggests a higher fidelity of the attachment model to the properties of the “target” biological network. As can be seen from Table 4, fidelity of the degree distributions between grown and target networks is higher for downlink-based attachment mechanisms as compared to the attachment kernel method of Mayo et al. (2012).

**Table 4:** **Statistics for the difference between power-law exponents of candidate and target network’s degree distributions resulting from either the attachment kernel method reported in Mayo et al. (2012), or from the downlink attachment method reported here**.

Attachment probability	Networks														
	1			2			3			4			5		
	In	Out	Total	In	Out	Total	In	Out	Total	In	Out	Total	In	Out	Total
**Attachment kernel method**															
Linear	0.91 ± 0.6	0.94 ± 0.6	0.81 ± 0.6	0.25 ± 0.3	0.55 ± 0.2	0.18 ± 0.1	0.86 ± 0.4	0.74 ± 0.3	0.63 ± 0.6	1.18 ± 0.5	0.87 ± 0.4	0.75 ± 0.7	0.8 ± 0.5	1.92 ± 0.2	0.21 ± 0.3
Power-law	1.09 ± 0.5	1.08 ± 0.5	0.99 ± 0.7	0.23 ± 0.2	0.57 ± 0.2	0.16 ± 0.1	0.8 ± 0.4	0.71 ± 0.4	0.73 ± 0.7	1.09 ± 0.5	0.99 ± 0.2	0.46 ± 0.6	0.88 ± 0.6	1.91 ± 0.2	0.19 ± 0.4
Sigmoidal	0.92 ± 0.6	0.98 ± 0.5	0.97 ± 0.7	0.42 ± 0.3	0.63 ± 0.1	0.15 ± 0.1	1.01 ± 0.5	0.66 ± 0.3	0.82 ± 0.6	1.25 ± 0.5	0.65 ± 0.4	1.09 ± 0.6	0.62 ± 0.5	1.91 ± 0.2	0.3 ± 0.2
**Downlink attachment method**															
Target attachment	0.08 ± 0.1	0.96 ± 0.6	0.13 ± 0.1	0.38 ± 0.0	0.21 ± 0.3	0.07 ± 0.1	0.62 ± 0.5	0.12 ± 0.1	0.1 ± 0.0	0.22 ± 0.2	0.44 ± 0.3	0.07 ± 0.0	1.89 ± 0.0	1.9 ± 0.0	0.35 ± 0.3
Substrate attachment	0.16 ± 0.1	1.4 ± 0.2	0.61 ± 0.4	0.37 ± 0.0	0.69 ± 0.2	0.02 ± 0.0	0.38 ± 0.0	0.9 ± 0.0	0.36 ± 0.6	0.48 ± 0.7	0.94 ± 0.2	0.37 ± 0.3	1.9 ± 0.0	1.9 ± 0.0	0.41 ± 0.4

**Table 5:** **Statistics or the difference between fitted power-law exponent for candidate and target networks’ distributions of genes participating in downlinks**.

Attachment probability	Networks				
	1	2	3	4	5
**Attachment kernel method**					
Linear	1.17 ± 0.5	1.19 ± 0.5	0.79 ± 0.4	1.32 ± 0.4	0.33 ± 0.3
Power-law	0.9 ± 0.5	1.27 ± 0.6	0.72 ± 0.1	1.07 ± 0.5	0.51 ± 0.2
Sigmoidal	1.43 ± 0.0	1.1 ± 0.3	0.86 ± 0.1	1.56 ± 0.1	0.15 ± 0.1
**Downlink attachment method**					
Target attachment	0.67 ± 0.2	0.43 ± 0.1	0.34 ± 0.0	0.75 ± 0.4	0.63 ± 0.6
Substrate attachment	0.75 ± 0.3	1.2 ± 0.5	0.34 ± 0.0	0.69 ± 0.4	0.62 ± 0.6

Error bounds for the distribution of nodes participating in downlink substructures show similar traits to that observed for the degree distributions. Out of the five substrates, the fifth network had marginally better distributions when grown with single node attachments for the same reasons explained above. Additionally, using the probabilities calculated from the target network (i.e., “target attachment, Tables 4 and 5) does not always lead to higher fidelity, as can be seen in the fourth and fifth networks. This is again because quite a few nodes do not participate in downlink structures and hence the probability distributions from the goal network make the counts skewed.

### 4.2 Evolutionary Mechanisms and Downlink-Based Network Growth

Preferential attachment mechanisms have been suggested, sometimes in addition to other mechanisms (e.g., duplication events), as models of evolutionary formation of gene-regulatory (Chung et al., 2003), protein interaction (Eisenberg and Levanon, 2003), and metabolic networks (Light et al., 2005). For gene-regulatory networks, mutations to DNA bases may alter the affinity of DNA-binding proteins or cis-regulatory modules to result in rewiring or admission of novel regulatory interactions (Erwin and Davidson, 2009). It is plausible that evolutionary mutations to DNA sequences result in creation of whole downlink transcriptional modules over a single generation, given the local nature of cis-regulatory mutation mechanisms and the potential for gene duplication events. For example, base-pair mutations can alter the availability of new binding sites, which manipulates the “distance” between interacting sites via insertion or deletion of cis-regulatory modules or sub-functionalization due to regional duplications, among others (Erwin and Davidson, 2009). At the system level, correlations between mutations over successive generations may be needed to consistently evolve new cis-regulatory modules and gene-regulatory interactions. However, even a node-by-node attachment mechanism (i.e., DNA sequence mutations that result in a single novel gene-regulatory interaction) holds potential for multiple novel gene-regulatory interactions formed over a single generation (Chung et al., 2003), which may explain the fewer nodes in the GRN observed to not participate in downlink modules. This can be linked to the error bounds generated for the fifth substrate, where results are marginally better for single node attachments; in this network only approximately 80% of the nodes participated in downlink motifs as opposed to ≥90% for networks labeled 1–4.

It is currently difficult to directly test hypotheses regarding network “growth” mechanisms due to experimental difficulties in manipulating the evolution of transcriptional networks in microorganisms such as bacteria. An attempt to experimentally emulate the “bottom up” approach employed in many attachment or duplication-based network growth mechanisms, such as the motif-based attachment method proposed in this paper, may be therefore impractical with current technologies. One alternative might be to reverse the growth process. Transcriptional regulatory networks, such as the *E. coli* network dataset analyzed here, serve as target states of the growth mechanisms; beginning with these fully formed networks and sequentially “deactivating” regulatory interactions between genes and transcription factors may provide valuable insight into the processes that formed them. For example, protein production could be suppressed with RNAi tailored to specific mRNA, thereby eliminating a regulatory interaction by preventing protein proliferation; another strategy could be to target a transcription factor’s activated state, perhaps by interfering with phosphorylation/dephosphorylation reactions through crosstalk (Rowland and Deeds, 2014), thus modulating its binding affinity to the correct DNA sequence and preventing gene activation. As a proof of principle, some experimental efforts have already succeeded in extensively “rewiring” *E. coli*’s transcriptional regulatory network (Isalan et al., 2008). Even so, future work is needed to predict dynamical consequences of adding or removing regulatory interactions specific to the attachment mechanism (in our case, regulatory interactions associated with downlink motifs), which could be evaluated using these or other experimental methods.

Recent developments in “*in vitro*” circuit design using microfluidic cell-free systems for the rapid prototyping of synthetic genetic networks as a “biomolecular breadboard” for molecular programing (OpenWetWare, 2014) is another promising avenue for experimentally validating the network growth principles proposed here. The biomolecular breadboards project has successfully synthesized different types of feed-forward loop motifs (Sen et al., 2014) and can be extended to design coupled FFL circuits. Similarly, such synthetic biology circuits of coupled downlink motifs can experimentally validate the dynamical consequences of our proposed network growth method thereby creating new hypotheses on whether coupled downlinks exhibit any preferences in natural selection. Currently however, this can only be achieved at a smaller scale by synthesizing small networks of connected downlinks.

## 5 Conclusion

We have presented a directed transcriptional network growing algorithm using the concept of motif-based preferential attachment, which allows for several new genes and regulatory interactions to be accumulated per step in the network evolution. While many existing algorithms in this area grow undirected networks using the preferential attachment model, or directed networks using the modified preferential attachment scheme with various attachment kernels, they fail to generate networks with high fidelity of motif distributions when contrasted with real-world biological networks. We have proposed using entire transcriptional motifs, which some view as “building blocks of complex networks” (Alon, 2007), as the fundamental unit of network evolution, rather than the accumulation of single genes and regulatory interactions at each potential growth opportunity. Our resulting networks built using this method exhibit higher fidelity to *E. coli* transcriptional networks, both in terms of degree distributions and downlink distributions.

Our algorithm accounts not only for the abundance of downlink motifs, which seem to cover most of the nodes and edges from the *E. coli* transcription regulatory network, but also accounts for two classes of nodes in gene-regulatory networks: genes and transcription factors. One interesting line of future work will be to understand how other transcriptional motifs and types of coupling may contribute to the overall properties of an evolved network model. Another possibility is to consider various centrality measures based on a network renormalized using VMN-based graph transformations. Nevertheless, realistic models of gene-regulatory network evolution will serve to aid future investigations into diverse phenomena, from dynamical signaling over transcriptional-regulatory networks to efforts relating network topology with biological function.

## Author Contributions

PG and MM conceptualized the study; AFA and AFM implemented the algorithm and obtained results. PG, MM, AFA, AFM, and EP helped in writing the manuscript.

## Conflict of Interest Statement

The authors declare that the research was conducted in the absence of any commercial or financial relationships that could be construed as a potential conflict of interest.
